# Assessing the impact of supervised interval training on cardiovascular autonomic neuropathy in type 2 diabetes patients

**DOI:** 10.14814/phy2.70476

**Published:** 2025-08-13

**Authors:** Laura Stirane, Karlis Stirans, Leonora Pahirko, Janis Mednieks, Karina Ostrovska, Aija Kļavina, Leo Selavo, Jelizaveta Sokolovska

**Affiliations:** ^1^ Faculty of Medicine and Life Sciences University of Latvia Riga Latvia; ^2^ Faculty of Residency Riga Stradins University Riga Latvia; ^3^ Faculty of Science and Technology University of Latvia Riga Latvia; ^4^ Faculty of Medicine, Department of Neurology and Neurosurgery Riga Stradins University Riga Latvia; ^5^ Laboratory of Sports and Nutrition Research Riga Stradins University Riga Latvia

**Keywords:** autonomic neuropathy, diabetes mellitus, Ewing score, exercise, interval walking

## Abstract

This post hoc analysis of the “Healthy walk” study evaluated the effect of interval walking training on cardiovascular autonomic neuropathy (CAN) in type 2 diabetes (T2D) patients. At baseline, 64 T2D patients underwent tilt table testing with autonomic reflex tests: heart rate responses to the Valsalva maneuver, deep breathing, and standing, and blood pressure responses to standing and sustained handgrip, using the Ewing score. Fifty‐six participants were allocated to an interval training (IT) group and control group. The IT group completed supervised interval walking training three times weekly for 60 min over 4 months, while the control group received physical activity education. Twenty‐four participants in the IT group and 30 in the control group completed the study. The primary endpoint of this post hoc analysis was the change in Ewing score. CAN was detected in 42 patients (66%), mean Ewing score of 2.7 ± 0.72. Those with CAN, 16 were in the IT group and 19 in the control group. Both groups showed a significant reduction in Ewing scores (IT: from 2.5 to 1.77, *p* = 0.003; control: from 2.72 to 1.91, *p* = 0.001). Interval walking training and physical activity education both reduce CAN severity in T2D patients.

## 1 INTRODUCTION

The growing global burden of type 2 diabetes (T2D) poses an enormous public health challenge. No longer confined to the elderly, nearly half of all diabetes cases now emerge in individuals in their fourth to fifth decades of life (Hu et al., 2015). One serious complication of diabetes is cardiovascular autonomic neuropathy (CAN), which results from damage to autonomic nerve fibers innervating the heart and blood vessels. This impairment, driven by hyperglycemia, leads to abnormalities in heart rate (HR) regulation and vascular dynamics (Vinik et al., 2003).

Clinically, CAN manifests as tachycardia, exercise intolerance, and orthostatic hypotension, and it is strongly associated with an increased risk of cardiovascular mortality. Despite being a common complication of diabetes, the significance of CAN has often been underappreciated.

A key method for investigating autonomic dysfunction is cardiovascular autonomic reflex tests (CARTs) using a tilt table. These tests evaluate HR and blood pressure (BP) responses to provocative physiological maneuvers: HR response to the Valsalva maneuver, deep breathing, and standing, and BP response to standing and a sustained handgrip. According to the Toronto Consensus, CARTs remain the gold standard for autonomic testing and are recognized by neurological scientific societies (Serhiyenko & Serhiyenko, 2018; Spallone, 2019). Despite the growing recognition of CAN's significant impact on diabetes patients, treatment options for CAN remain extremely limited and lack sufficient evidence supporting their efficacy, with no unified treatment algorithms currently. Intensive glycemic management has been shown to reduce CAN prevalence in type 1 diabetes, while a multifactorial approach—encompassing glycemic control, BP management, weight loss, diet, and physical activity—may be more effective in T2D. Some medications have demonstrated promising results in addressing the manifestations of CAN, including metformin, alpha‐lipoic acid, angiotensin‐converting enzyme inhibitors and angiotensin II receptor blockers, beta‐blockers, fluvastatin, sodium‐glucose cotransporter‐2 inhibitors, and glucagon‐like peptide‐1 receptor agonists. Additionally, physical activity plays a crucial role in the treatment of CAN (Eleftheriadou et al., 2024).

Lifestyle interventions have beneficial effects on autonomic function in prediabetes. For example, in a study involving 2980 prediabetes patients, lifestyle intervention positively affected measures of cardiovascular autonomic function (HR, heart rate variability (HRV), and QT interval length) (Carnethon et al., 2006).

Previous research has demonstrated that different forms of physical activity, including low‐to‐moderate intensity aerobic activities like walking or running, induce measurable improvements in cardiac autonomic regulation, including enhanced HRV (Fisher & Tahrani, 2017). Moreover, interval walking training is a suitable mode of endurance physical activity for patients with T2D due to its accessibility and tolerability. Several studies have shown that moderate‐intensity interval training is effective in improving autonomic function (Colberg et al., 2010; Voulgari et al., 2013). Previously, we have reported positive effects of interval walking training on albuminuria and leptin/adiponectin ratio, underlying its beneficial influence on the cardiovascular system and insulin sensitivity (Sokolovska et al., 2020).

In individuals with T2D, a study evaluated the effects of 4 months of aerobic and resistance training on cardiovascular autonomic function—specifically heart rate variability and baroreflex sensitivity—in 30 patients without cardiovascular autonomic neuropathy (CAN). The study found that both aerobic and resistance training improved autonomic function, with aerobic training demonstrating superior results (Bellavere et al., 2018). To summarize, there is some promising data regarding the effects of accessible physical activity modes on CAN in individuals with T2D. However, further studies are needed to develop effective physical activity‐based approaches for managing CAN in T2D.

The work aimed to assess cardiovascular autonomic neuropathy (CAN) in individuals with type 2 diabetes and evaluate the impact of interval walking training, delivered via mobile devices, as part of the “Healthy Walk” study.

## 2 MATERIALS AND METHODS

### 2.1 Recruitment, screening, physical activity intervention

Here, we report the post hoc analysis of the study “Healthy Walk” which involved subjects with T2D allocated to interval walking training managed through smart mobile devices or standard lifestyle education. The primary endpoint of this post hoc analysis was defined as the change in the Ewing score from baseline to study completion, reflecting the impact of the intervention on the severity of cardiovascular autonomic neuropathy (CAN) in patients with type 2 diabetes. The study was conducted in Riga, Latvia, from October 2017 to November 2018. The study was in line with the 1975 Declaration of Helsinki and received approval Nr 1‐03/17 (30.06.2017) of the Scientific Research Ethics Committee of the Institute of Cardiology and Regenerative Medicine of the University of Latvia.

A detailed description of the study “Healthy Walk” is reported in Sokolovska et al. (2020). In brief, 78 physically inactive patients with T2D aged 35–75 years were recruited for the study. Exclusion criteria included increased exercise‐induced cardiovascular risk (per stress ECG results), pre‐existing cardiovascular disease (unstable angina, myocardial infarction, coronary or leg artery stents/shunts, stroke, and claudication), chronic kidney disease (stage IV–V), insulin therapy, regular structured physical activity (>150 min/week), severe visual impairment, severe retinopathy or macular edema, severe musculoskeletal disorders of the legs, severe peripheral neuropathy, or disabling somatic or psychiatric conditions, and pregnancy. Baseline leisure‐time physical activity was quantified in terms of Metabolic Equivalent of Task (MET) units. Activities were assigned MET values according to the Compendium of Physical Activities, and total energy expenditure was calculated in MET‐minutes by multiplying the MET value by the activity duration in minutes (Ainsworth et al., 2011). A VO_2_ peak test (walking with incremental incline) was performed on a Life Fitness treadmill until volitional fatigue, using a modified Bruce protocol. Of the 78 initially screened patients, 14 subjects were excluded due to the exclusion criteria. Sixty‐four patients underwent a tilt table test for CAN assessment. Eight patients refused to participate in the intervention after the baseline visit.

A statistician performed the randomization using R software to create two groups with similar characteristics, including diabetes duration, age, gender distribution, HbA1c, lipid levels, body mass index, and VO_2_ peak measurement. Fifty‐six participants continued the study and were allocated into the interval training group (*n* = 26) and the control group (*n* = 30). The IT group followed 4 months of interval walking training, consisting of three sessions per week, each lasting 60 min. Each session began with a 5‐min warm‐up at a self‐selected walking speed, gradually increasing from low‐to‐moderate intensity. The main phase of the session lasted 50 min and consisted of alternating 3‐min intervals of low‐ and moderate‐intensity walking at 40% and 70% of peak energy expenditure, respectively. The session concluded with a 5‐min cooldown period at a self‐selected low intensity walking pace. Participants in the interval training group used smartphones equipped with a custom application, *Instawalk*, which allowed researchers to remotely monitor the intervention. The participants in the control group received a structured education session with recommendations to adhere to international physical activity guidelines (Colberg et al., 2016), engaging in at least 150 min per week of structured physical activity.

**FIGURE 1:**
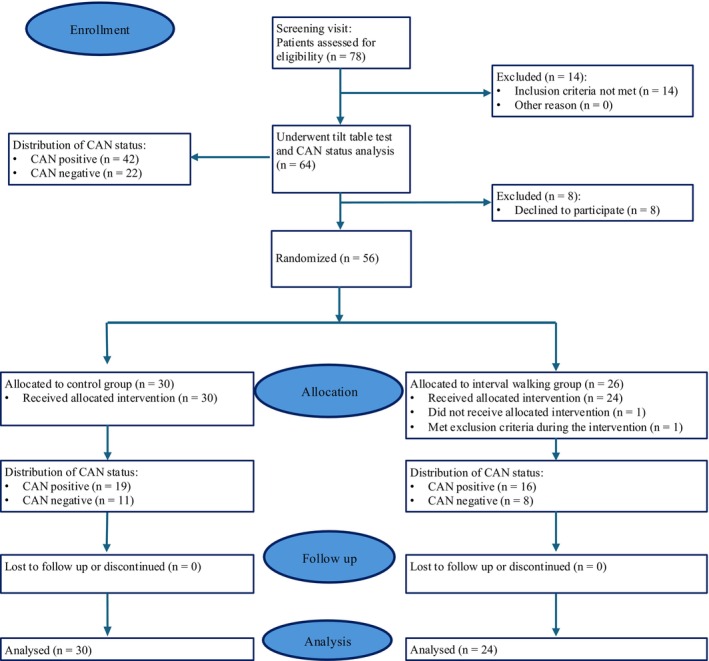
This flowchart outlines the design of a study evaluating the impact of physical activity on cardiovascular autonomic neuropathy (CAN) in patients with type 2 diabetes. Seventy‐eight patients were assessed for eligibility; 14 patients were excluded according to exclusion criteria. At baseline, 64 patients underwent tilt table test using cardiovascular autonomic reflex tests. There were 42 CAN‐positive and 22 CAN‐negative patients in our cohort. Then 56 subjects were allocated to an interval training group (IT) (*n* = 26) and to a control group (*n* = 30). There were 16 CAN‐positive and 8 CAN‐negative patients in the IT group. There were 19 CAN‐positive and 11 CAN‐negative patients in the control group. A total of 24 IT and 30 control participants underwent pre‐ and post‐intervention evaluation of CAN.

Tilt table test was repeated after 4 months (*n* = 54). Two patients from the IT group were excluded: one because of a violation of protocol (did not perform any training sessions) and another one due to the commencement of insulin therapy. See the trial flow chart in Figure 1.

### 2.2 Assessment of cardiovascular autonomic nervous system: Tilt table tests and Ewing score

Each participant underwent tilt table test before and after the intervention to determine the degree of damage to the autonomic nervous system, the presence of CAN, and changes in autonomic function. CAN was evaluated using five standard CARTs proposed by the Ewing battery. Three tests assessed parasympathetic function: HR response to the Valsalva maneuver, deep breathing, and standing. Two tests assessed sympathetic function: BP response to standing and a sustained handgrip (Ewing & Clarke, 1982). Continuous electrocardiogram and BP had been recorded throughout all tests. CARTs values (classified as normal, borderline, and abnormal) are presented in Table 1, along with their interpretation.

**TABLE 1:** References of cardiovascular autonomic reflex tests (Ewing & Clarke, 1982, Lin et al., 2017).

Cardiovascular autonomic reflex test	Measurement	Normal value (0 points)	Borderline value (0.5 points)	Abnormal value (1 points)
1. Heart rate response to the Valsalva maneuver	Valsalva ratio, mm	≥ 1.21	1.11–1.20	≤ 1.10
2. Heart rate response to standing	30 s/15 s ratio, mm	≥ 1.04	1.01–1.03	≤ 1.00
3. Heart rate response to deep breathing	Expiration/Inspiration (E/I) ratio, beats/min	≥ 15	11–14	≤ 10
4. Blood pressure response to standing	Fall in systolic blood pressure, mmHg	≤ 10	11–29	≥ 30
5. Blood pressure response to a sustained handgrip	Rise in diastolic blood pressure, mmHg	≥ 16	11–15	≤ 10

#### 2.2.1 Heart rate response to the Valsalva maneuver (Valsalva ratio)

The participant was instructed to blow into a mouthpiece connected to an aneroid manometer and maintain a pressure of 40 mmHg for 15 s, repeated three times. During the strain period, BP decreases, while HR increases. In healthy individuals, BP rises above its resting value, and HR slows after the strain period. In contrast, in patients with autonomic damage, BP gradually declines during the strain period and returns to the normal range slowly after release, without overshoot in BP or a corresponding HR change. The Valsalva ratio is calculated as the longest R‐R interval after the maneuver divided by the shortest R‐R interval during the maneuver (Ewing et al., 1985; Ewing & Clarke, 1982).

#### 2.2.2 Heart rate response to deep breathing (E/I ratio)

**FIGURE 2:**
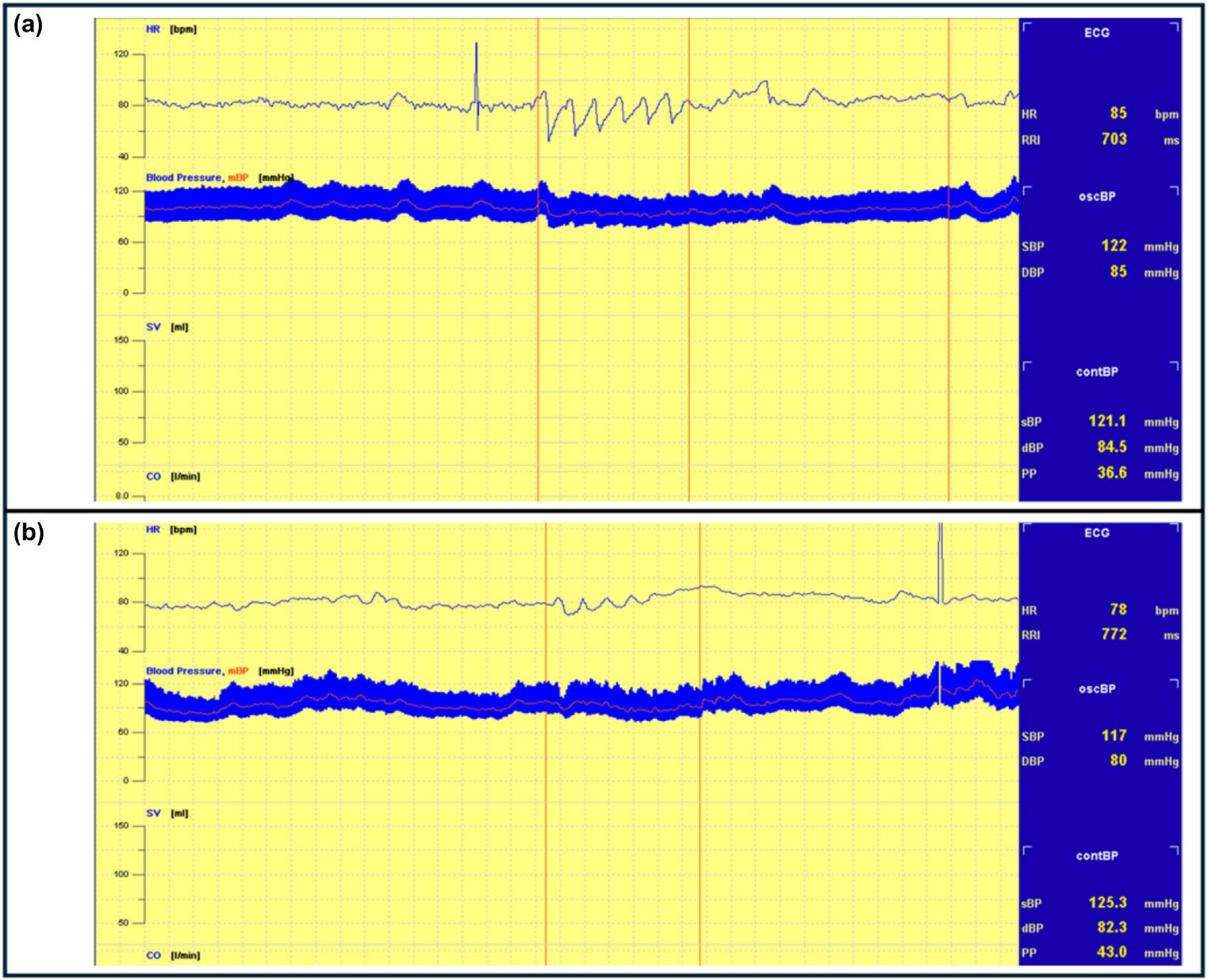
The heart rate response to deep breathing: (a) normal finding, (b) abnormal finding. Examples were taken from the participants in this study.

The patient was instructed to breathe deeply at a rate of six cycles per minute, with 5 s for inspiration and 5 s for expiration. This breathing pattern was recorded for 1 min. In healthy individuals, heart rate varies continuously, whereas in patients with autonomic neuropathy, HR variation is either reduced or completely absent; there is a complete absence or noticeable reduction. The result was expressed as the mean difference between maximum and minimum HR across the six cycles, calculated as the expiration/inspiration (E/I) ratio (Ewing et al., 1985; Ewing & Clarke, 1982). The HR response to deep breathing is illustrated in Figure 2 (a—normal finding and b—decreased rate variation in case of autonomic damage).

#### 2.2.3 Heart rate response to standing (30 s/15 s ratio)

The patient was lying down in a supine position for 5 min on a tilt table, and then the table was elevated to 70 degrees. During these changes in healthy subjects, a characteristic immediate rapid increase in heart rate occurs with a maximum at the 15th beat, and a relative overshoot bradycardia occurs with a maximum at the 30th beat. In the case of autonomic neuropathy, there is an absence or gradual increase in heart rate after standing. HR response is expressed by the 30 s/15 s ratio, which is the shortest R‐R interval (15th beat) to the longest R‐R interval (30th beat) after standing (Ewing et al., 1985; Ewing & Clarke, 1982).

#### 2.2.4 Blood pressure response to standing (fall in systolic blood pressure)

The patient lay supine on a tilt table for 5 min, after which BP was measured. The table was then elevated to 70 degrees, and another BP reading was recorded. The difference between the systolic blood pressure (SBP) in the supine position and the SBP in the standing position was used to assess the postural fall in SBP. In healthy individuals, standing causes blood pooling in the lower extremities, leading to a temporary drop in SBP, which is rapidly corrected by peripheral vasoconstriction. However, in patients with autonomic neuropathy, SBP decreases upon standing and remains lower than in the supine position on the tilt table. A fall in SBP greater than 30 mmHg is considered indicative of severe autonomic damage (Ewing et al., 1985; Ewing & Clarke, 1982).

#### 2.2.5 Blood pressure response to a sustained handgrip (rise in diastolic blood pressure)

A handgrip dynamometer was used to determine the maximum contraction. Participants were then instructed to maintain 30% of their achieved maximum contraction for as long as possible, up to 5 min. BP was measured three times before the handgrip and at 1‐min intervals during the task. In healthy individuals, diastolic blood pressure (DBP) rises sharply during sustained handgrip. However, in cases of autonomic neuropathy, this increase is abnormally small due to extensive peripheral sympathetic dysfunction. The difference between the highest DBP recorded during the exercise and the mean DBP before the handgrip was used as the outcome measure expressed as the result (rise in DBP) (Lin et al., 2017).

#### 2.2.6 Ewing score

Each test was assessed with a score: 0—normal, 0.5—borderline, and 1—abnormal. The sum of these 5 scores made up the Ewing score, which was used to assess the severity of CAN. Ewing score ≥2 was classified as CAN positive, and Ewing score less than 2 was classified as CAN negative (Lin et al., 2017).

In the end, both the IT and control groups were stratified into CAN‐positive and CAN‐negative patients. The IT group consisted of 16 CAN‐positive and 8 CAN‐negative patients, and the control group included 19 CAN‐positive and 11 CAN‐negative patients.

### 2.3 Statistical analysis

All statistical analysis was performed using statistical software R and R Studio version 4.4.0. (Team, 2024) (last accessed on the 28th of December 2024). Quantitative variables were expressed as mean values with standard deviations, and differences between the two groups were tested using Student's *t*‐test. Categorical variables were represented as counts and percentages; the chi‐square test was used to test the equality of proportions. The impact of interval walking training on CARTs was tested using two‐way repeated measures ANOVA models adjusted for sex and diabetes duration (using R package *afex* (Singmann et al., 2015)). The mean differences between the control and interval training groups and between CAN‐negative versus CAN‐positive patients at baseline correspond to the main between‐group effects. The pre‐ and post‐measurements were considered as a main within‐group effect. Post hoc pairwise comparison of the estimated marginal means of simple between‐group and within‐group effects was carried out using the Tukey *p* value adjustment method (using R package *emmeans* (Russell et al., 2024)).

## 3 RESULTS

### 3.1 Characteristics of subjects and assessment of CAN status at baseline

At baseline, 64 T2D subjects underwent the CAN testing. The mean age of the participants was 58.62 ± 9.55 years, with a mean duration of diabetes of 6.84 ± 5.12 years. Participants were obese; the body mass index was 33.46 ± 5.48 kg/m^2^, but their diabetes was well controlled (HbA1c 6.87 ± 1.25% and fasting glucose 7.56 ± 2.47 mmol/L). 73.44% of patients had arterial hypertension. The mean low‐density lipoprotein level was 3.19 mmol/L. The mean estimated glomerular filtration rate (eGFR) was 139.14 ± 49.3 mL/min/1.73 m^2^. According to metabolic equivalents, our participants were moderately active in daily activities (MET 33.72 ± 25.19) (Table S1).

According to the criteria of Ewing score, 42 out of 64 patients (66%) were classified as CAN‐positive. The mean Ewing score for all participants (*n* = 64) at baseline was more than 2 points (Ewing score 2.16 ± 1.01), which indicates that there was a tendency to autonomic dysfunction in our cohort. Analyzing our patients regarding the Ewing score we conclude that CAN‐positive patients had no severe autonomic damage on average (mean 2.7 ± 0.72 points). Severe CAN was detected in four patients according to postural SBP fall of more than 30 mmHg (BP response to standing test) (data not shown). There were significant differences in Ewing score between CAN‐positive and CAN‐negative patients (2.7 ± 0.72 vs. 1.11 ± 0.53 points, *p* < 0.001) (Table S1).

CAN‐positive patients had a statistically significantly higher eGFR of 147.74 ± 55.82 mL/min/1.73m^2^, compared to CAN‐negative subjects, whose eGFR was 122.73 ± 28.32 mL/min/1.73m^2^ (*p* = 0.021) (Table S1). Otherwise, there were no statistically significant differences in anthropometric, clinical (including duration of diabetes), or laboratory data between CAN‐positive and CAN‐negative patients at baseline (Table S1). Analyzing all patients, four out of five CARTs (except BP response to a sustained handgrip) on average were determined as borderline according to the reference values (Table 1) for the standardized cardiac autonomic function tests. The mean values of these CARTs were as follows: Valsalva ratio, 1.11 ± 0.09; 30 s/15 s ratio, 1.03 ± 0.08; E/I ratio, 12.17 ± 4.37 beats per minute; and the fall in SBP after standing, 11.92 ± 11.31 mmHg (Table S1).

Evaluating each CART separately between CAN‐positive versus CAN‐negative patients, there were statistically significant differences in the following tests: HR response to Valsalva maneuver (Valsalva ratio: 1.09 ± 0.07 vs. 1.15 ± 0.11 mm, *p* = 0.028); HR response to standing (30 s/15 s ratio: 1.01 ± 0.05 vs. 1.07 ± 0.1 mm, *p* = 0.012); HR response to deep breathing (E/I ratio: 10.41 ± 3.56 vs. 15.45 ± 3.86 beats/min, *p* < 0.001); BP response to standing (fall in SBP: 14.81 ± 11.9 vs. 6.41 ± 7.67 mmHg, *p* = 0.001). However, there were no statistically significant differences between CAN‐positive and CAN‐negative participants in BP response to a sustained handgrip test (rise in DBP: 25.43 ± 13.48 vs. 26.59 ± 8.4 mmHg, *p* = 0.673) (Table S1). All participants were able to hold the handgrip dynamometer for 1 min. Of these, 58 participants could maintain the grip for 2 min, 20 participants for 3 min, and none for 5 min.

According to the references of CARTs, CAN‐positive patients mean results were in the borderline range for three tests (HR response to standing (30 s/15 s ratio: 1.01 ± 0.05 mm), HR response to deep breathing (E/I ratio: 10.41 ± 3.56 beats/min), and BP response to standing (fall in SBP: 14.81 ± 11.9 mmHg)). One test was in the abnormal range (HR response to the Valsalva maneuver (Valsalva ratio: 1.09 ± 0.07 mm)) and one test was in the normal range (BP to a sustained handgrip (rise in DBP: 25.43 ± 13.48 mmHg)) (Table S1).

CAN‐negative participants showed that four out of five tests were in the normal range (HR response to standing (30 s/15 s ratio: 1.07 ± 0.1 mm), HR response to deep breathing (E/I ratio: 15.45 ± 3.86 beats/min), BP response to standing (fall in SBP: 6.41 ± 7.67 mmHg), and BP response to a sustained handgrip (rise in DBP: 26.59 ± 8.4 mmHg)), and one test was in the borderline range (HR response to the Valsalva maneuver (Valsalva ratio: 1.15 ± 0.11 mm)) (Table S1).

Clinical characteristics and results of CARTs in patients stratified by CAN status are shown in Table S1.

### 3.2 Impact of interval walking on cardiovascular autonomic reflex tests

Clinical characteristics of the IT and the control group are presented in Table 2. There were no significant differences in the anthropometric data of the patients between the IT and the control groups. The mean Ewing score did not differ between groups. The duration of diabetes was statistically significantly longer in the IT group (see Table 2).

**TABLE 2:** Clinical characteristics of the interval training and the control groups before intervention.

Variable	All (*n* = 54)	Control group (*n* = 30)	Interval training group (*n* = 24)	*p*
**Demographic data**				
Gender: male/female, *n* (%)	18/36 (33/67)	7/23 (23/77)	11/13 (46/54)	0.146
Age, years	59.41 ± 9.57	60 ± 8.78	58.67 ± 10.61	0.623
Weight, kg	91.83 ± 18.08	88.7 ± 16.99	95.75 ± 18.98	0.162
Body mass index, kg/m^2^	32.81 ± 5.14	32.69 ± 5.42	32.97 ± 4.88	0.845
Waist/hip ratio	0.96 ± 0.08	0.94 ± 0.07	0.97 ± 0.09	0.144
Systolic blood pressure, mmHg	133.35 ± 15.11	131.27 ± 14.74	135.96 ± 15.48	0.264
Diastolic blood pressure, mmHg	82.54 ± 7.91	81.70 ± 6.38	83.58 ± 9.54	0.412
Arterial hypertension, *n* (%)	39 (72)	20 (67)	19 (79)	0.476
Heart rate, beats/min	73.56 ± 7.45	73.47 ± 8.61	73.67 ± 5.88	0.920
Duration of diabetes, years	7.26 ± 5.37	5.80 ± 4.29	9.08 ± 6.09	**0.031**
Metabolic equivalents, MET	34.57 ± 26.15	36.89 ± 28.9	31.67 ± 22.51	0.459
Non‐proliferative diabetic retinopathy, *n* (%)	4 (7.5)	3 (10)	1 (4.2)	> 0.99
Diabetic peripheral neuropathy, *n* (%)	5 (9.3)	2 (6.7)	3 (12.5)	> 0.99
Diabetic nephropathy, *n* (%)	5 (9.3)	3 (10)	2 (8.3)	> 0.99
Metformin, *n* (%)	46 (85.2)	27 (90.0)	19 (79.2)	0.467
Sulfonylurea, *n* (%)	15 (27.8)	7 (23.3)	8 (33.3)	0.610
DPP4 inhibitors, *n* (%)	12 (22.2)	5 (16.7)	7 (29.2)	0.442
GLP‐1 agonists, *n* (%)	4 (7.4)	1 (3.3)	3 (12.5)	0.450
SGLT‐2 inhibitors, *n* (%)	5 (9.3)	1 (3.3)	4 (16.7)	> 0.99
Antihypertensive drugs, *n* (%)	36 (66.7)	19 (63.3)	17 (70.8)	0.772
Statins, *n* (%)	16 (29.6)	12 (40.0)	4 (16.7)	0.117
eGFR, mL/min/1.73 m^2^	131.63 ± 42.46	124.97 ± 44.32	139.96 ± 39.35	0.194
HbA1c, %	6.76 ± 1.12	6.7 ± 1.18	6.85 ± 1.07	0.636
Fasting glucose, mmol/L	7.30 ± 2.32	7.00 ± 2.33	7.68 ± 2.30	0.291
Total cholesterol, mmol/L	5.19 ± 1.21	5.12 ± 1.31	5.27 ± 1.09	0.645
HDLC, mmol/L	1.33 ± 0.37	1.33 ± 0.32	1.33 ± 0.42	0.968
LDLC, mmol/L	3.10 ± 1.11	3.20 ± 1.14	2.97 ± 1.06	0.457
Triglycerides, mmol/L	2.29 ± 3.59	1.63 ± 0.65	3.12 ± 5.28	0.182
VO_2_ peak relative, mL/kg/min	22.66 ± 5.54	21.21 ± 4.08	24.11 ± 6.48	0.110
**Cardiovascular autonomic reflex tests results and Ewing score**				
**1. Heart rate response to the Valsalva maneuver (Valsalva ratio), mm**	1.12 ± 0.09	1.11 ± 0.10	1.13 ± 0.08	0.288
Shortest R‐R interval during the Valsalva maneuver, mm	0.65 ± 0.08	0.66 ± 0.09	0.63 ± 0.07	0.189
Longest R‐R interval after the Valsalva maneuver, mm	0.72 ± 0.10	0.73 ± 0.10	0.72 ± 0.11	0.697
**2. Heart rate response to standing (30s/15s ratio), mm**	1.03 ± 0.08	1.04 ± 0.09	1.0 ± 0.06	0.559
Shortest R‐R interval at the 15th beat, mm	0.73 ± 0.10	0.7 ± 0.10	0.73 ± 0.09	0.597
Longest R‐R interval at the 30th beat, mm	0.75 ± 0.10	0.77 ± 0.11	0.74 ± 0.08	0.288
**3. Heart rate response to deep breathing (Expiration/Inspiration ratio), beats/min**	12.52 ± 4.48	12.23 ± 4.85	12.88 ± 4.04	0.598
**4. Blood pressure response to standing (Fall in SBP), mmHg**	10.96 ± 9.32	10.13 ± 9.52	12.00 ± 9.16	0.468
**5. Blood pressure response to a sustained handgrip (Rise in DBP), mmHg**	25.70 ± 11.2	24.57 ± 11.12	27.12 ± 11.38	0.411
**Ewing score, points**	2.10 ± 0.98	2.15 ± 1.07	2.04 ± 0.87	0.683

Table S2 presents the full results from the repeated measures ANOVA for CARTs and the Ewing score, adjusted for sex and diabetes duration. Post hoc pairwise group comparison results are summarized in Table 3 and described in the subsequent paragraphs.

**TABLE 3:** Impact of interval training on cardiovascular autonomic reflex tests.

Variable	CAN	Control group (*n* = 30)			Interval training group (*n* = 24)		
		Pre‐Con	Post‐Con	*p*‐value Pre‐Con vs Post‐Con	Pre‐IT	Post‐IT	*p*‐value Pre‐IT vs Post‐IT
**1.Valsalva ratio, mm**	Negative	1.15 (1.09, 1.21)	1.16 (1.08, 1.24)	0.759	1.14 (1.07, 1.21)	1.25 (1.15, 1.35)	0.077
	Positive	1.08 (1.03, 1.13)	1.17 (1.1, 1.23)	**0.031**	1.12 (1.07, 1.16)	1.25 (1.19, 1.32)	**0.001**
*p*‐value CAN+ vs CAN−		0.054	0.965		0.577	0.94	
Shortest RR during the Valsalva maneuver, mm	Negative	0.65 (0.6, 0.7)	0.64 (0.59, 0.69)	0.671	0.65 (0.58, 0.71)	0.62 (0.56, 0.68)	0.477
	Positive	0.67 (0.63, 0.72)	0.65 (0.61, 0.69)	0.366	0.62 (0.58 ,0.67)	0.64 (0.6, 0.68)	0.553
*p‐*value CAN+ vs CAN−		0.511	0.711		0.510	0.649	
Longest R‐R interval after the Valsalva maneuver, mm	Negative	0.74 (0.67, 0.8)	0.74 (0.69, 0.8)	0.839	0.74 (0.66, 0.82)	0.77 (0.7, 0.84)	0.475
	Positive	0.73 (0.67, 0.78)	0.76 (0.71, 0.81)	0.200	0.7 (0.64, 0.75)	0.79 (0.75, 0.84)	**0.001**
*p‐*value CAN+ vs CAN−		0.788	0.626		0.388	0.574	
**2. 30s/15s ratio, mm**	Negative	1.05 (1.01, 1.08)	1.05 (1.01, 1.08)	0.986	1.06 (1.02, 1.10)	1.06 (1.02, 1.10)	0.98
	Positive	1.01 (0.98, 1.04)	1.07 (1.04, 1.09)	**0.003**	1.01 (0.98, 1.04)	1.08 (1.05, 1.10)	**< 0.001**
*p‐*value CAN+ vs CAN−		0.094	0.397		0.051	0.475	
Shortest R‐R interval at the 15th beat, mm	Negative	0.75 (0.69, 0.82)	0.76 (0.69, 0.84)	0.820	0.71 (0.63, 0.79)	0.78 (0.69, 0.88)	0.158
	Positive	0.73 (0.68, 0.79)	0.75 (0.69, 0.82)	0.582	0.73 (0.67, 0.78)	0.78 (0.72, 0.84)	0.11
*P‐*value CAN+ vs CAN−		0.636	0.851		0.689	0.975	
Longest R‐R interval at the 30th beat, mm	Negative	0.80 (0.74, 0.86)	0.79 (0.72, 0.86)	0.755	0.75 (0.67, 0.82)	0.78 (0.7, 0.87)	0.444
	Positive	0.75 (0.69, 0.8)	0.81 (0.75, 0.87)	**0.038**	0.72 (0.67, 0.77)	0.85 (0.8, 0.91)	**< 0.001**
*p‐*value CAN+ vs CAN−		0.149	0.675		0.589	0.178	
**3. Expiration/Inspiration ratio, beats/min**	Negative	17.3 (14.91, 19.69)	16.74 (14.12, 19.36)	0.596	15.02 (11.99, 18.05)	12.41 (9.09, 15.73)	0.057
	Positive	9.87 (8.01, 11.72)	8.99 (6.96, 11.03)	0.289	11.68 (9.84, 13.52)	12.28 (10.26, 14.3)	0.462
*p‐*value CAN+ vs CAN−		**< 0.001**	**< 0.001**		0.062	0.947	
**4. Fall in SBP after standing, mmHg**	Negative	6.94 (1.16, 12.72)	7.31 (1.38, 13.25)	0.921	7.37 (0.18, 14.56)	13.1 (5.71, 20.48)	0.220
	Positive	11.84 (7.14, 16.53)	13.24 (8.41, 18.06)	0.644	14.31 (9.65, 18.98)	15.16 (10.37, 19.95)	0.779
*p‐*value CAN+ vs CAN−		0.180	0.115		0.108	0.638	
**5. Rise in DBP during a sustain handgrip, mmHg**	Negative	23.96 (17.24, 30.68)	26.09 (20.56, 31.61)	0.585	26.69 (18.28, 35.11)	28.57 (21.65, 35.5)	0.700
	Positive	26.46 (20.47, 32.45)	26.96 (22.03, 31.89)	0.885	25.54 (20.12, 30.96)	21.47 (17.01, 25.93)	0.199
*p*‐value CAN+ vs CAN−		0.565	0.806		0.816	0.087	
**Ewing score, points**	Negative	1.14 (0.71, 1.57)	1.33 (0.87, 1.78)	0.525	1.09 (0.55, 1.62)	1.2 (0.63, 1.76)	0.760
	Positive	2.72 (2.37, 3.06)	1.91 (1.54, 2.28)	**0.001**	2.5 (2.15, 2.84)	1.77 (1.41, 2.14)	**0.003**
*p*‐value CAN+ vs CAN−		**< 0.001**	**0.043**		**< 0.001**	0.090	

#### 3.2.1 Heart rate response to the Valsalva maneuver

**FIGURE 3:**
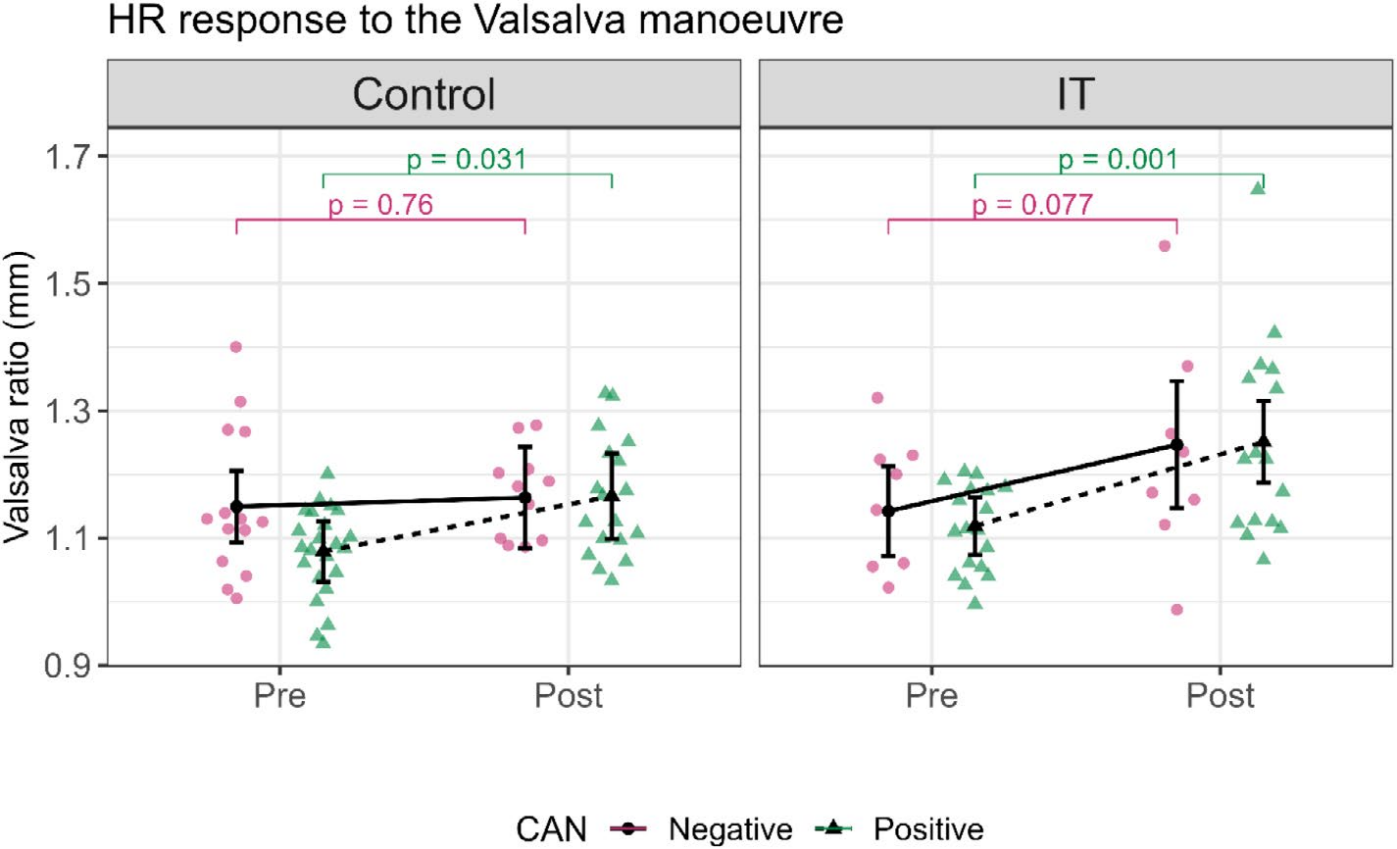
This figure shows the influence of the intervention on heart rate (HR) response to the Valsalva maneuver test in control and interval training (IT) groups. HR response to the Valsalva maneuver is expressed as the Valsalva ratio, measured as the longest R‐R interval after the maneuver to the shortest R‐R interval during the maneuver. Control and IT are divided into Pre and Post conditions, showing the data before and after the intervention. CAN: cardiac autonomic neuropathy, pink circles, and continuous lines represent CAN‐negative patients, and green triangles and dotted lines represent CAN‐positive patients. In the control group: The CAN‐negative patients show minimal change from Pre to Post (*p* = 0.76), and the CAN‐positive patients show a statistically significant increase (*p* = 0.031). In the IT group: The CAN‐positive patients show a highly significant increase (*p* = 0.001), and the CAN‐negative patients show a tendency to statistically significant increase from Pre to Post (*p* = 0.077).

In both the IT and control groups, CAN‐positive patients showed a statistically significant improvement in the Valsalva ratio after the intervention. In the IT group, the Valsalva ratio of CAN‐positive patients changed from borderline to normal range, with pre‐intervention values of 1.12 (1.07, 1.16) and post‐intervention values of 1.25 (1.19, 1.32) (*p* = 0.001). In the control group, CAN‐positive patients' Valsalva ratio improved from abnormal to borderline range, with pre‐intervention values of 1.08 (1.03, 1.13) and post‐intervention values of 1.17 (1.01, 1.23) (*p* = 0.031). In the CAN‐negative participants of the IT group, the Valsalva ratio showed improvement from pre‐intervention values of 1.14 (1.07, 1.21) to post‐intervention values of 1.25 (1.15, 1.35) (*p* = 0.077) (Figure 3, Table 3).

#### 3.2.2 Heart rate response to standing

**FIGURE 4:**
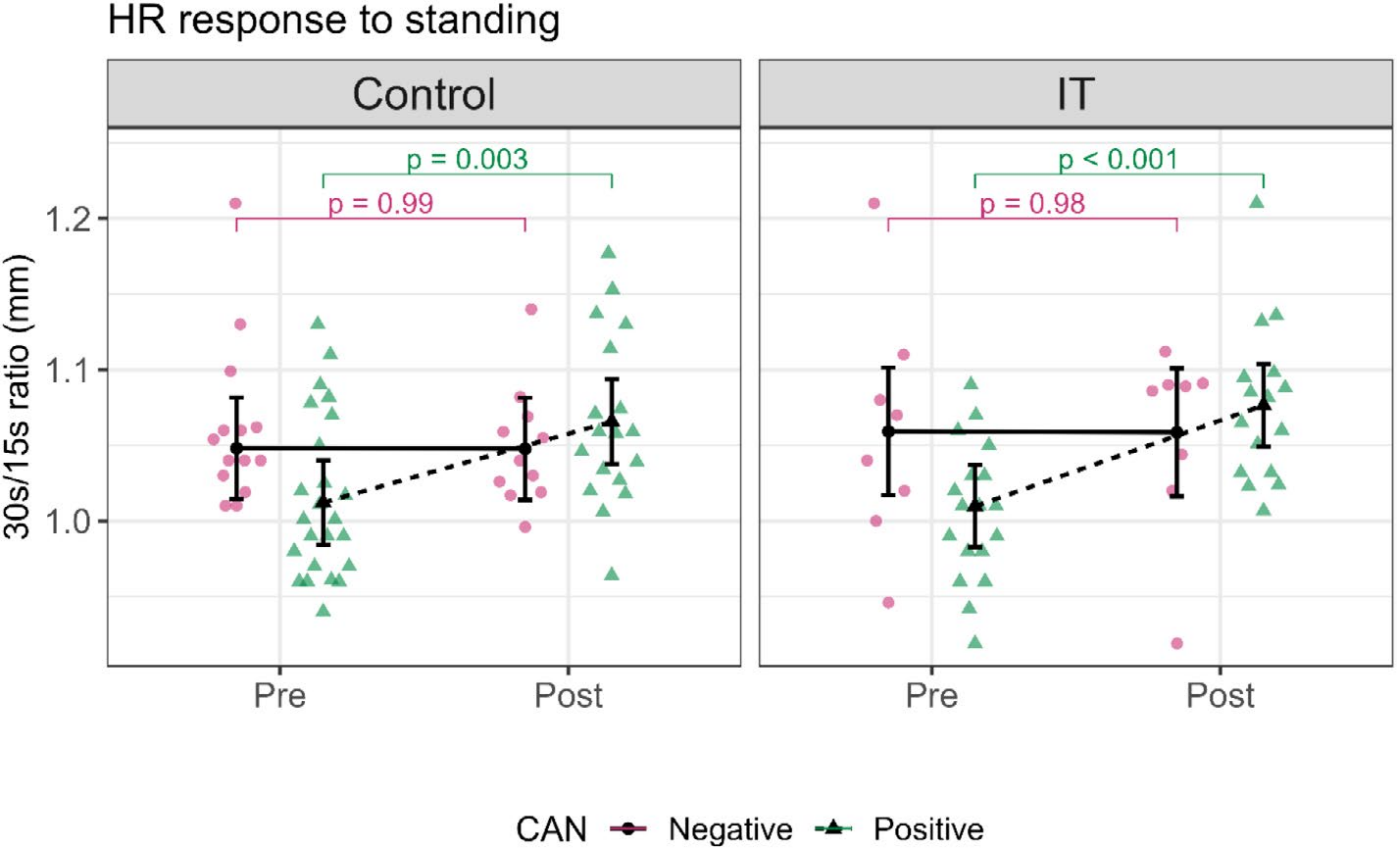
This figure shows the influence of the intervention on heart rate (HR) response to standing test in control and interval training (IT) groups. The HR response to standing is expressed as the 30 s/15 s ratio, measured as the shortest R‐R interval (15th beat) to the longest R‐R interval (30th beat) after standing. Control and IT are divided into Pre and Post conditions, showing the data before and after the intervention. CAN: Cardiac autonomic neuropathy, pink circles, and continuous lines represent CAN‐negative patients, and green triangles, and dotted lines represent CAN‐positive patients. In the control group: The CAN positive patients show a statistically significant increase (*p* = 0.003), and the CAN negative patients show minimal change from Pre to Post (*p* = 0.99). In the IT group: The CAN‐positive patients show a highly significant increase (*p* < 0.001), and the CAN‐negative patients show minimal change from Pre to Post (*p* = 0.98).

In the IT group, CAN‐positive patients showed a statistically significant improvement in their 30 s/15 s ratio, changing from borderline to normal values. The 30 s/15 s ratio increased from 1.01 (0.98, 1.04) pre‐intervention to 1.08 (1.05, 1.10) post‐intervention (*p* < 0.001). In the control group, CAN‐positive patients also showed a statistically significant improvement in the 30 s/15 s ratio, from borderline to normal values, with a change in the 30 s/15 s ratio from 1.01 (0.98, 1.04) pre‐intervention to 1.07 (1.04, 1.09) post‐intervention (*p* = 0.003). In both groups, the 30 s/15 s ratio of CAN‐negative patients did not change during the intervention (Figure 4, Table 3).

#### 3.2.3 Heart rate response to deep breathing

**FIGURE 5:**
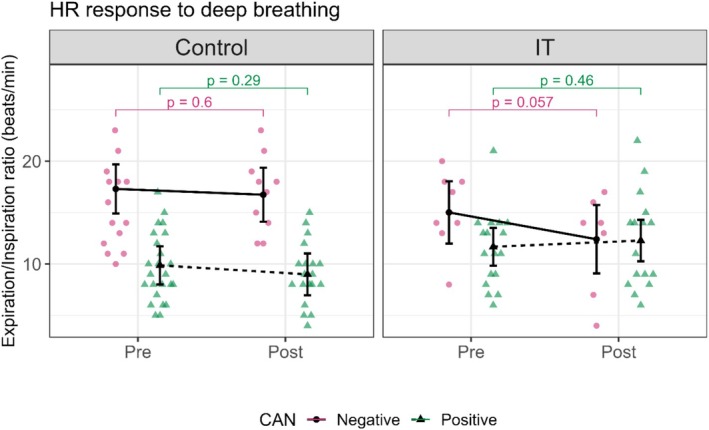
This figure shows the influence of the intervention on heart rate (HR) response to deep breathing test in control and interval training (IT) groups. HR response to deep breathing is expressed as the E/I ratio, measured as the mean of the difference between the maximum and minimum heart rate of the six cycles of deep breathing. Control and IT are divided into Pre and Post conditions, showing the data before and after the intervention. CAN: cardiac autonomic neuropathy, pink circles, and continuous lines represent CAN‐negative patients, and green triangles, and dotted lines represent CAN‐positive patients. In the control group: The CAN‐positive and negative patients show minimal change from Pre to Post (*p* = 0.60 and *p* = 0.29). In the IT group: The CAN‐positive patients show minimal changes Pre to Post (*p* = 0.46), and CAN‐negative patients show a tendency to statistically significant changes Pre to Post (*p* = 0.057).

There were no statistically significant changes in the E/I ratio in any of the groups. However, a positive tendency was observed in the IT group among CAN‐positive patients, where the E/I ratio increased from 11.68 (9.84, 13.52) pre‐intervention to 12.28 (10.26, 14.3) post‐intervention (*p* = 0.462). In contrast, in the control group, CAN‐positive patients showed no changes in the E/I ratio, with values remaining: 9.87 (8.01, 11.72) pre‐intervention and 8.99 (6.96, 11.03) post‐intervention (*p* = 0.289) (Figure 5, Table 3).

#### 3.2.4 Blood pressure response to standing

**FIGURE 6:**
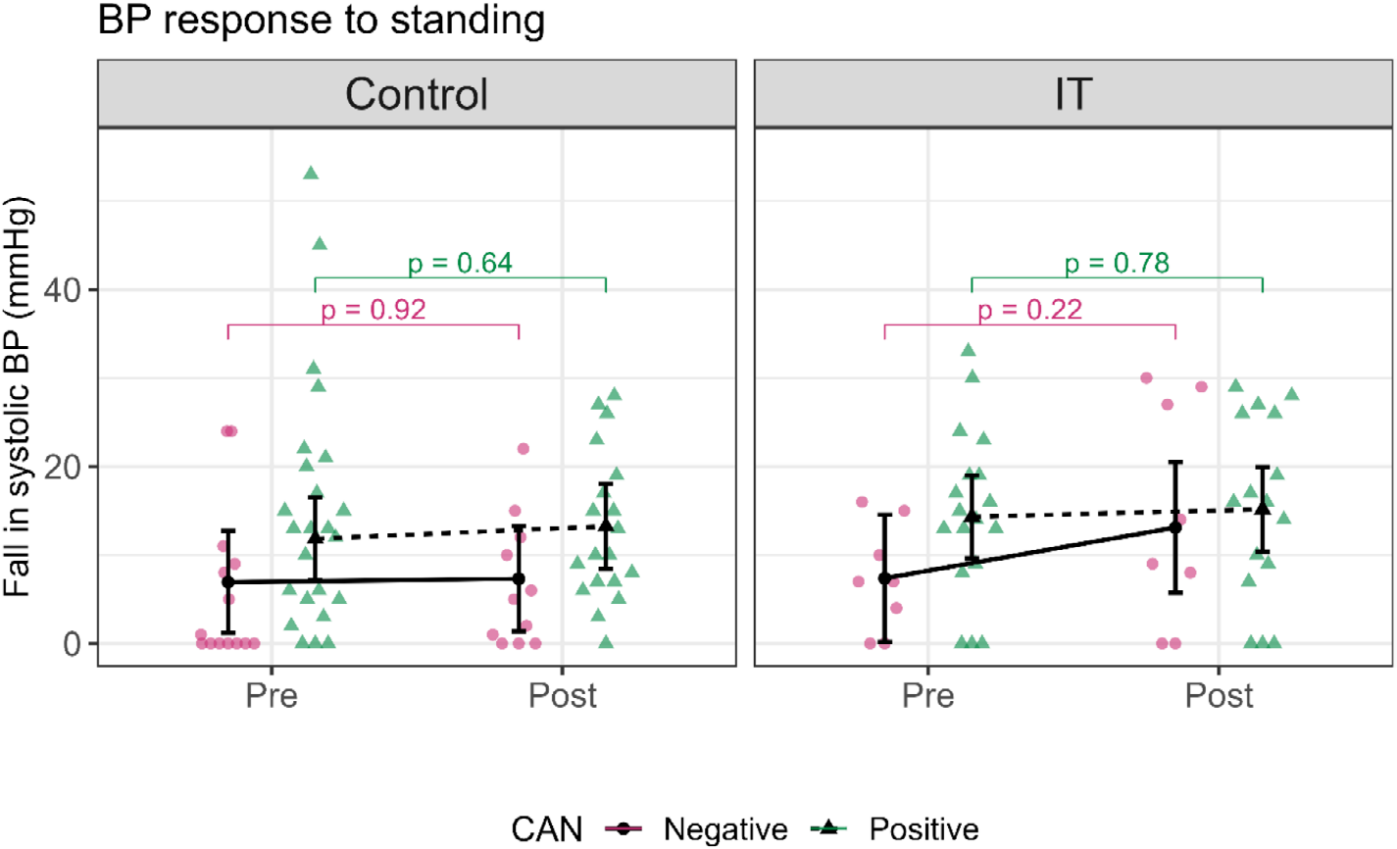
This figure shows the influence of the intervention on blood pressure (BP) response to standing test in control and interval training (IT) groups. BP response to standing is expressed as a fall in systolic blood pressure (SPB), measured as the difference between the systolic BP lying and the systolic BP standing. Control and IT are divided into Pre and Post conditions, showing the data before and after the intervention. CAN: cardiac autonomic neuropathy, pink circles, and continuous lines represent CAN‐negative patients, and green triangles, and dotted lines represent CAN‐positive patients. In the control group: The CAN positive and negative patients show minimal change from Pre to Post (*p* = 0.92 and *p* = 0.64). In the IT group: The CAN positive and negative patients show minimal change from Pre to Post (*p* = 0.22 and *p* = 0.78).

There were no statistically significant changes in the fall of SBP after verticalization in any of the groups. In CAN‐positive patients of both study groups, borderline values were observed before and after intervention. The IT group showed a fall in SBP from 14.31 (9.65, 18.98) pre‐intervention to 15.16 (10.37, 19.95) (*p* = 0.779), while the control group showed a fall in SBP from 11.84 (7.14, 16.53) pre‐intervention to 13.24 (8.41, 18.06) post‐intervention (*p* = 0.644) (Figure 6, Table 3).

#### 3.2.5 Blood pressure response to a sustained handgrip

**FIGURE 7:**
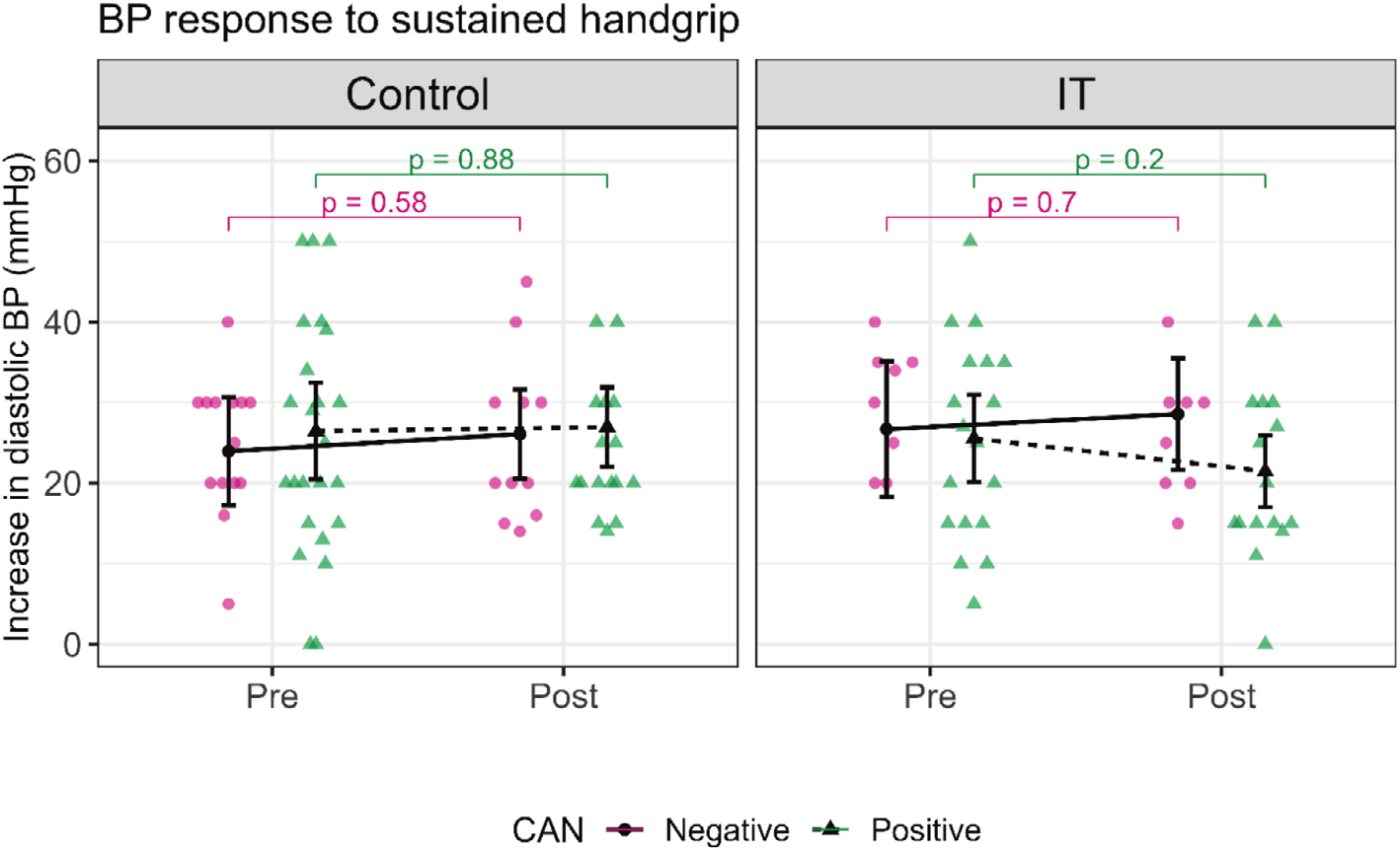
This figure shows the influence of the intervention on blood pressure (BP) response to a sustained handgrip test in control and interval training (IT) groups. BP response to a sustained handgrip is expressed as a rise in diastolic blood pressure (DPB), measured as the difference between the highest DBP during the test and the mean DBP before handgrip. Control and IT are divided into Pre and Post conditions, showing the data before and after the intervention. CAN: cardiac autonomic neuropathy, pink circles, and continuous lines represent CAN‐negative patients, and green triangles, and dotted lines represent CAN‐positive patients. In the control group: The CAN positive and negative patients show minimal change from Pre to Post (*p* = 0.58 and *p* = 0.88). In the IT group: The CAN positive and negative patients show minimal change from Pre to Post (*p* = 0.70 and *p* = 0.20).

The rise in DBP values was within the normal values among all participants. In the IT group, CAN‐positive patients showed a rise in DBP from 25.54 (20.12, 30.96) pre‐intervention to 21.47 (17.01, 25.93) post‐intervention (*p* = 0.199), while in the control group, CAN‐positive patients had a rise in DBP from 26.46 (20.47, 32.45) pre‐intervention to 26.96 (22.03, 31.89) post‐intervention (*p* = 0.885) (Figure 7, Table 3).

#### 3.2.6 Changes in CAN severity during the intervention (Ewing score)

**FIGURE 8:**
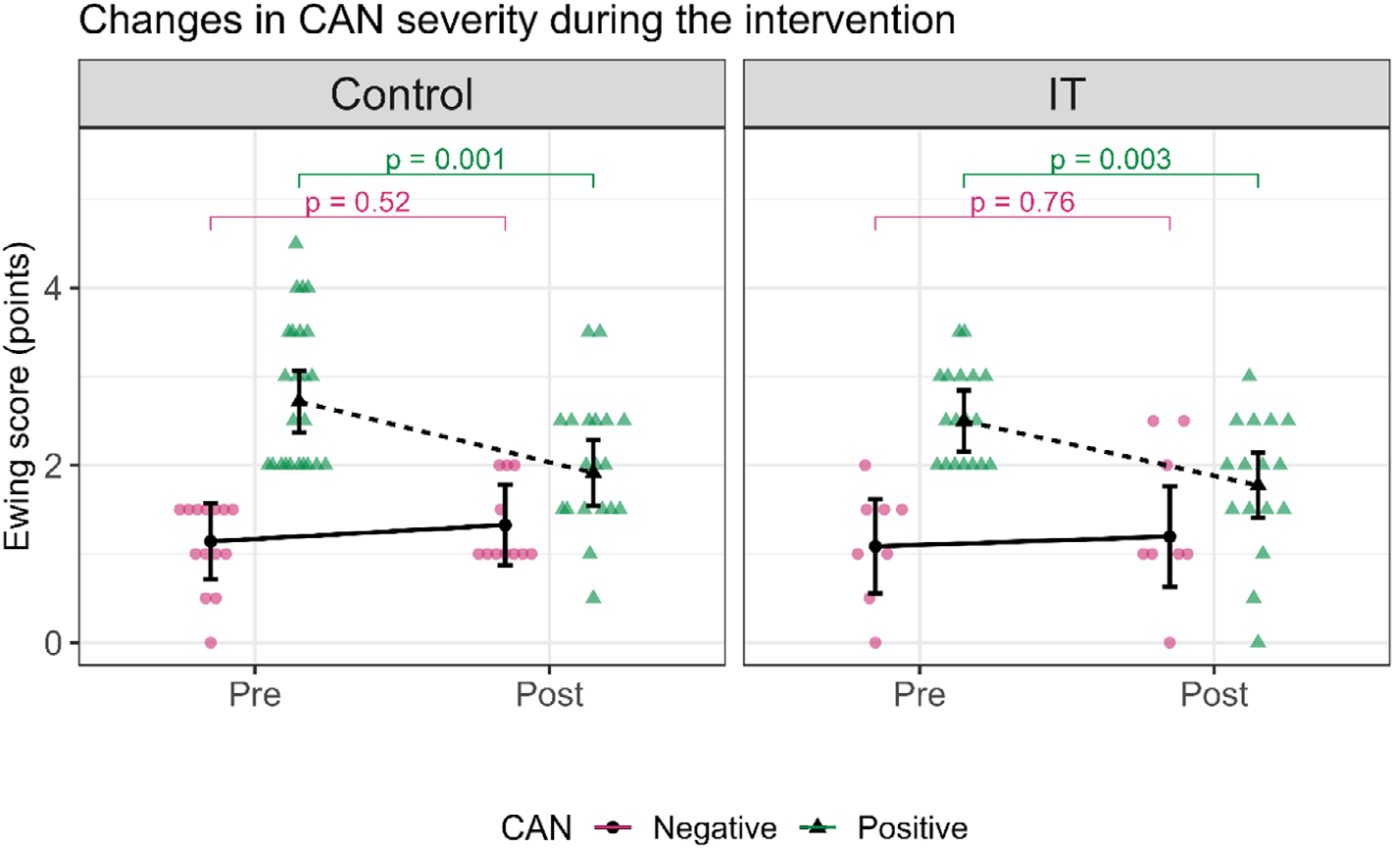
This figure shows the influence of the intervention on CAN severity, as indicated by the Ewing score, which was calculated based on five cardiovascular autonomic reflex tests, in the control and interval training group. CAN: cardiac autonomic neuropathy, pink circles, and continuous lines represent CAN‐negative patients, and green triangles, and dotted lines represent CAN‐positive patients. In the control group: The CAN‐negative group shows a slight, nonsignificant increase in the Ewing score from Pre to Post (*p* = 0.52), CAN‐positive group shows a significant decrease in the Ewing score from Pre to Post (*p* = 0.001). In the IT group: The CAN‐negative group shows a relatively stable Ewing score with no significant changes from Pre to Post (*p* = 0.76), CAN‐positive group shows a statistically significant decrease in the Ewing score from Pre to Post (*p* = 0.003).

A statistically significant reduction in the Ewing score was observed for CAN‐positive patients in both the IT and control groups. In the IT group, the Ewing score decreased from 2.5 (2.15, 2.84) pre‐intervention to 1.77 (1.41, 2.14) post‐intervention (*p* = 0.003), while in the control group, the Ewing score decreased from 2.72 (2.37, 3.06) pre‐intervention to 1.91 (1.54, 2.28) post‐intervention (*p* = 0.001). After the intervention, CAN‐positive patients in both groups showed mean values equivalent to those of CAN‐negative patients. No significant changes in the Ewing score were observed in the CAN‐negative patients. In the IT group, the Ewing score remained unchanged from 1.09 (0.55, 1.62) pre‐intervention to 1.20 (0.63, 1.76) post‐intervention (*p* = 0.760), and in the control group, the Ewing score remained unchanged from 1.14 (0.71, 1.57) pre‐intervention to 1.33 (0.87, 1.78) post‐intervention (*p* = 0.525) (Figure 8, Table 3).

## 4 DISCUSSION

In our cohort, the prevalence of CAN among patients with T2D was 66%, which is slightly higher than the rates previously reported in the literature. Studies have documented a CAN prevalence ranging from 20% to 40% in T1D and 25% to 50% in T2D after 20 years of disease duration. The higher prevalence observed in our cohort may be attributed to prolonged periods of undiagnosed hyperglycemia, which can accelerate the onset and progression of diabetes‐related complications, including CAN (Freeman et al., 2011). Although the average reported duration of diabetes in our cohort was under 10 years, it is plausible that the condition remained undiagnosed and untreated for a longer time, thereby increasing cumulative glycemic burden. Moreover, our patients exhibited multiple cardiometabolic risk factors, such as obesity, hypertension, and dyslipidemia, which may have contributed to an earlier onset or greater severity of autonomic dysfunction. Additionally, the considerable variation in reported CAN prevalence across studies may be due to inconsistencies in diagnostic criteria and methodologies used to assess CAN. This study focused on evaluating the status and severity of CAN using the tilt table test and CARTs before and after interval walking training or physical activity recommendations in patients with T2D.

The diagnosis of CAN is challenging due to complex tests, varying criteria, and limited research, making it a frequently unrecognized diabetes complication. Diagnoses rely on a combination of clinical assessments, symptom evaluation, and specific autonomic function tests; for instance, a higher score on the Autonomic Symptom Questionnaire (COMPASS 31) is indicative of more severe autonomic symptoms (Eleftheriadou et al., 2024). Various approaches can be used to assess, including prolonged QT and reduced HRV detected via ECG and 24‐h Holter monitoring (Eleftheriadou et al., 2024; Lin et al., 2017), non‐dipping or reverse dipping BP pattern identified through ambulatory BP monitoring (Eleftheriadou et al., 2024), sudomotor dysfunction assessment (Ziemssen & Siepmann, 2019), and corneal nerve loss detected via corneal confocal microscopy (Serhiyenko & Serhiyenko, 2018; Spallone, 2019). Myocardial scintigraphy and baroreflex sensitivity testing, due to their specialized and costly nature, are primarily reserved for research settings. The definitive diagnosis should continue to rely on CARTs, as recommended by various guidelines (Eleftheriadou et al., 2024; Stevens, 2001). Preferably, autonomic function tests should be noninvasive, safe, easy to perform, reproducible, sensitive, specific, and suitable for long‐term studies. Although the tilt table test and CARTs remain the gold standard for CAN evaluation, they are complex, time‐consuming, and require specialized equipment (Eleftheriadou et al., 2024). At baseline, patients diagnosed with CAN demonstrated borderline results in three tests: the HR response to standing, the HR response to deep breathing, and the BP response to standing. Additionally, they showed abnormal values in the HR response to the Valsalva maneuver test but normal values in the BP response to the sustained handgrip test. The accuracy of these tests for diagnosis has been evaluated in several studies in patients with CAN. Among CAN‐positive patients, the HR response to deep breathing and the HR response to the Valsalva maneuver are most frequently abnormal, indicating significant autonomic dysfunction. The HR response to standing is often borderline, reflecting early autonomic impairment in CAN‐positive patients (Vinik et al., 2003; Vinik & Ziegler, 2007). In contrast, the BP response to the sustained handgrip test is usually within normal limits in CAN‐positive patients, as it assesses sympathetic function, which may be preserved for a longer duration (Körei et al., 2017).

In our study, four patients demonstrated severe autonomic involvement, as indicated by a SBP drop of more than 30 mmHg in the BP response to the standing test. However, based on the Ewing score, CAN‐positive patients, on average, did not demonstrate severe autonomic impairment. When evaluating the results of each CART between CAN‐positive and CAN‐negative patients, we observed that the BP response to the sustained handgrip test was the only parameter that did not show a statistically significant difference between them. This can be explained by several factors. First, many patients were unable to maintain sustained handgrip for the duration required by the CART criteria. Additionally, the test was difficult to perform. The relevance of the sustained handgrip test in CAN assessment has been debated. Körei et al. evaluated its association with the other four CARTs and concluded that the BP response to the sustained handgrip test should no longer be included in the CARTs, as its results are highly dependent on baseline DBP (Körei et al., 2017). In comparison, only one patient in our study was unable to perform the HR response to the Valsalva maneuver test, while another could not complete the HR response to the deep breathing test due to dizziness. Despite this, the HR response to the deep breathing test is widely regarded as a simple and highly sensitive test for assessing the cardiovagal parasympathetic cholinergic function and requires minimal patient cooperation (Shields, 2009).

We found that CAN is associated with hyperfiltration, which contributes to the development of diabetic nephropathy. In our study, CAN positive patients had statistically significantly higher GFR, indicating kidney involvement (Oh et al., 2020). The association between CAN and renal hyperfiltration has been investigated in previous research. Increased sympathetic nervous system activity, which is commonly observed in CAN patients, may contribute to renal vasoconstriction, leading to hyperfiltration (Moriconi et al., 2023). Apart from eGFR, there were no significant differences in anthropometric, clinical, or laboratory data at baseline between CAN positive and CAN negative patients.

The primary endpoint of the study was achieved, with a significant reduction in the Ewing score, indicating improvement in CAN among patients with positive CAN in both groups following the intervention. The main finding of our study was that 4 months of regular, moderate‐intensity interval walking training led to a statistically significant improvement in CAN severity. Specifically, the intervention resulted in a notable reduction in the Ewing score in participants with positive CAN, highlighting the potential benefits of structured exercise in managing CAN in type 2 diabetes patients. Before the intervention, the average Ewing score in the IT group exceeded two, whereas it dropped below two after the intervention. Notably, even though the control group received only education on physical activity without direct supervision, CAN‐positive participants still exhibited a significant reduction in the Ewing score, indicating an improvement in CAN severity. This effect was likely due to the participants' motivation to engage in the study and adherence to the recommended physical activity guidelines (150 min per week) (Colberg et al., 2016). The observed improvement in CAN severity in both groups may be attributed to the beneficial vascular effects of physical activity. In contrast, CAN‐negative participants in both groups demonstrated no significant change in CARTs results.

Previous studies demonstrated that physical exercise positively influences autonomic nervous system function in otherwise healthy elderly individuals and patients with T2D. Exercise training has been shown to reduce cardiac sympathetic activity and enhance peripheral vasodilation (Dantas et al., 2015; Madden et al., 2006; Sarmento et al., 2017; Schuit et al., 1999). Aerobic exercise has also been associated with improvement in sympathovagal balance for cardiac patients (Besnier et al., 2017). Cassidy et al. examined the impact of high‐intensity interval training on CAN in T2D patients, demonstrating positive changes in glycemia but no significant improvements in cardiac autonomic function (Cassidy et al., 2019). In contrast, other studies indicated that moderate‐intensity aerobic exercise may improve autonomic function in TD2 patients (Colberg et al., 2010; Voulgari et al., 2013). More recently, systematic reviews have strengthened this evidence base. Bhati et al. reported that 15 out of 18 studies observed improvements in autonomic function after exercise interventions in T2D patients (Bhati et al., 2018). Picard et al. reviewed 21 studies and found that exercise significantly improved heart rate variability (HRV) in patients with type 2 diabetes. Improvements were attributed to reduced sympathetic and enhanced parasympathetic activity, with the greatest benefits seen in individuals with longer diabetes duration, dyslipidemia, and those engaged in supervised training (Picard et al., 2021). Hamasaki et al. conducted a systematic review of eight randomized controlled trials and found that exercise interventions, including aerobic and combined aerobic resistance training, led to beneficial effects on cardiovascular autonomic function in patients with T2D (Hamasaki, 2023). In our study, CAN‐positive patients showed significant improvement after the intervention in two of five CART tests: the HR response to the Valsalva maneuver and the HR response to standing, with values shifting from borderline to normal. This suggests a possible enhancement in parasympathetic activity following the intervention. While direct comparisons with previous studies are limited due to methodological differences in autonomic function assessment (for example, HRV vs. Ewing battery), our findings align with the broader literature indicating exercise‐induced benefits for autonomic regulation in T2D. Compared to Picard et al., who primarily used HRV measures (Picard et al., 2021), our study employed the Ewing battery and similarly found parasympathetic improvement, particularly in the HR response to the Valsalva maneuver and the HR response to standing tests, supporting the notion that exercise, even at moderate intensity, can restore autonomic balance in T2D patients.

Interestingly, a significant reduction in the overall Ewing score was also observed in the control group, which did not receive structured training but only received educational materials on physical activity. This unexpected finding may reflect increased awareness and behavioral change prompted by study participation, consistent with the Hawthorne effect often observed in clinical trials. Overall, our findings support the modifiability of CAN through lifestyle intervention in T2D patients and highlight the dual importance of structured exercise and patient education in promoting improvements in cardiac autonomic function.

Increased sympathetic activity is known to be associated with higher mortality, while exercise training can help preserve functional sympatholysis by enhancing blood flow distribution within active muscle through improved aerobic metabolism. In the elderly population, nitric oxide plays a crucial role in muscle‐induced vasodilation during exercise. Additionally, nitric oxide stimulates vagal modulation, inhibits sympathetic neurotransmission, and contributes to heart rate regulation through autonomic nervous system control or direct modulation of the sinoatrial node (Green et al., 2004). Regular physical activity also improves neurovascular control of muscle blood flow. The beneficial effects of exercise on vascular function include adaptations of blood vessels to the hemodynamic demands of training, reduced insulin resistance, and improved oxygen delivery (Sarmento et al., 2017).

Several limitations of this study should be acknowledged. To enhance the reliability of the findings, a larger study group, a longer duration of interval training, and greater adherence to the training protocol are needed. Additionally, not all CARTs were easily for patients to perform, especially BP response to a sustained handgrip test. A key strength of this study is the direct assessment of CAN severity before and after the intervention, enabling a clear evaluation of its impact of the intervention on autonomic function.

## 5 CONCLUSION

In conclusion, our findings highlight the effectiveness of interval walking training in improving CAN severity specifically in individuals with positive CAN, while demonstrating the stability of autonomic function in those without CAN. These results suggest that the intervention is both beneficial for patients with existing CAN and safe for those without autonomic dysfunction. The clinical impact of this study lies in demonstrating that interval walking training can reduce CAN severity, which is an independent risk factor for cardiovascular and cerebrovascular diseases in patients with T2D.

## FUNDING INFORMATION

Latvian State Genome database project; University of Latvia; Microtik donation administered by Foundation of the University of Latvia.

## CONFLICT OF INTEREST STATEMENT

The authors have no conflict of interest to declare.

## ETHICS STATEMENT

The study was in line with the 1975 Declaration of Helsinki and received approval Nr 1–03/17 (30.06.2017) of the Scientific Research Ethics Committee of the Institute of Cardiology and Regenerative Medicine of the University of Latvia.

## PATIENT CONSENT STATEMENT

Patients signed a consent form.

## Supporting information


Tables S1–S2.

## Data Availability

All data supporting the results are included in the manuscript and are available from the corresponding author upon reasonable request.

## References

[phy270476-bib-0001] Ainsworth, B. E. , Haskell, W. L. , Herrmann, S. D. , Meckes, N. , Bassett, D. R., Jr. , Tudor‐Locke, C. , Greer, J. L. , Vezina, J. , Whitt‐Glover, M. C. , & Leon, A. S. (2011). 2011 compendium of physical activities: A second update of codes and MET values. Medicine and Science in Sports and Exercise, 43, 1575–1581. 10.1249/MSS.0b013e31821ece12 21681120

[phy270476-bib-0002] Bellavere, F. , Cacciatori, V. , Bacchi, E. , Gemma, M. L. , Raimondo, D. , Negri, C. , Thomaseth, K. , Muggeo, M. , Bonora, E. , & Moghetti, P. (2018). Effects of aerobic or resistance exercise training on cardiovascular autonomic function of subjects with type 2 diabetes: A pilot study. Nutrition, Metabolism, and Cardiovascular Diseases, 28, 226–233. 10.1016/j.numecd.2017.12.008 29402509

[phy270476-bib-0003] Besnier, F. , Labrunée, M. , Pathak, A. , Pavy‐Le Traon, A. , Galés, C. , Sénard, J.‐M. , & Guiraud, T. (2017). Exercise training‐induced modification in autonomic nervous system: An update for cardiac patients. Annals of Physical and Rehabilitation Medicine, 60(1), 27–35. 27542313 10.1016/j.rehab.2016.07.002

[phy270476-bib-0004] Bhati, P. , Shenoy, S. , & Hussain, M. E. (2018). Exercise training and cardiac autonomic function in type 2 diabetes mellitus: A systematic review. Diabetes and Metabolic Syndrome: Clinical Research and Reviews, 12, 69–78. 10.1016/j.dsx.2017.08.015 28888482

[phy270476-bib-0005] Carnethon, M. R. , Prineas, R. J. , Temprosa, M. , Zhang, Z. M. , Uwaifo, G. , & Molitch, M. E. (2006). The association among autonomic nervous system function, incident diabetes, and intervention arm in the diabetes prevention program. Diabetes Care, 29, 914–919. 10.2337/diacare.29.04.06.dc05-1729 16567837 PMC1751934

[phy270476-bib-0006] Cassidy, S. , Vaidya, V. , Houghton, D. , Zalewski, P. , Seferovic, J. P. , Hallsworth, K. , Macgowan, G. A. , Trenell, M. I. , & Jakovljevic, D. G. (2019). Unsupervised high‐intensity interval training improves glycaemic control but not cardiovascular autonomic function in type 2 diabetes patients: A randomised controlled trial. Diabetes & Vascular Disease Research, 16, 69–76. 10.1177/1479164118816223 30541346 PMC6327303

[phy270476-bib-0007] Colberg, S. R. , Sigal, R. J. , Fernhall, B. , Regensteiner, J. G. , Blissmer, B. J. , Rubin, R. R. , Chasan‐Taber, L. , Albright, A. L. , & Braun, B. (2010). Exercise and type 2 diabetes: The American College of Sports Medicine and the American Diabetes Association: Joint position statement. Diabetes Care, 33, e147–e167. 10.2337/dc10-9990 21115758 PMC2992225

[phy270476-bib-0008] Colberg, S. R. , Sigal, R. J. , Yardley, J. E. , Riddell, M. C. , Dunstan, D. W. , Dempsey, P. C. , Horton, E. S. , Castorino, K. , & Tate, D. F. (2016). Physical activity/exercise and diabetes: A position statement of the American Diabetes Association. Diabetes Care, 39, 2065–2079. 10.2337/dc16-1728 27926890 PMC6908414

[phy270476-bib-0009] Dantas, F. F. , Cabral, T. G. , Silvestre, A. C. , Batista, R. M. , Santos, M. S. , & Santos, A. C. (2015). Effectiveness of leisure physical activities in vasodilatory response and blood pressure in middle‐aged and elderly women. JEP Online, 18, 66–77.

[phy270476-bib-0010] Eleftheriadou, A. , Spallone, V. , Tahrani, A. A. , & Alam, U. (2024). Cardiovascular autonomic neuropathy in diabetes: An update with a focus on management. Diabetologia, 67, 2611–2625. 10.1007/s00125-024-06242-0 39120767 PMC11604676

[phy270476-bib-0011] Ewing, D. J. , & Clarke, B. F. (1982). Diagnosis and management of diabetic autonomic neuropathy. British Medical Journal (Clinical Research Ed.), 285, 916–918. 10.1136/bmj.285.6346.916 6811067 PMC1500018

[phy270476-bib-0012] Ewing, D. J. , Martyn, C. N. , Young, R. J. , & Clarke, B. F. (1985). The value of cardiovascular autonomic function tests: 10 years experience in diabetes. Diabetes Care, 8, 491–498. 10.2337/diacare.8.5.491 4053936

[phy270476-bib-0013] Fisher, V. L. , & Tahrani, A. A. (2017). Cardiac autonomic neuropathy in patients with diabetes mellitus: Current perspectives. Diabetes, Metabolic Syndrome and Obesity: Targets and Therapy, 10, 419–434. 10.2147/dmso.S129797 29062239 PMC5638575

[phy270476-bib-0014] Freeman, R. , Wieling, W. , Axelrod, F. B. , Benditt, D. G. , Benarroch, E. , Biaggioni, I. , Cheshire, W. P. , Chelimsky, T. , Cortelli, P. , Gibbons, C. H. , Goldstein, D. S. , Hainsworth, R. , Hilz, M. J. , Jacob, G. , Kaufmann, H. , Jordan, J. , Lipsitz, L. A. , Levine, B. D. , Low, P. A. , … Van Dijk, J. G. (2011). Consensus statement on the definition of orthostatic hypotension, neurally mediated syncope and the postural tachycardia syndrome. Clinical Autonomic Research, 21, 69–72. 10.1007/s10286-011-0119-5 21431947

[phy270476-bib-0015] Green, D. J. , Maiorana, A. , O'driscoll, G. , & Taylor, R. (2004). Effect of exercise training on endothelium‐derived nitric oxide function in humans. The Journal of Physiology, 561, 1–25. 10.1113/jphysiol.2004.068197 15375191 PMC1665322

[phy270476-bib-0016] Hamasaki, H. (2023). The effect of exercise on cardiovascular autonomic nervous function in patients with diabetes: A systematic review. Healthcare (Basel), 11, 2668. 10.3390/healthcare11192668 37830705 PMC10572826

[phy270476-bib-0017] Hu, F. B. , Satija, A. , & Manson, J. E. (2015). Curbing the diabetes pandemic: The need for global policy solutions. JAMA, 313, 2319–2320. 10.1001/jama.2015.5287 25996138 PMC5291074

[phy270476-bib-0018] Körei, A. E. , Kempler, M. , Istenes, I. , Vági, O. E. , Putz, Z. , Horváth, V. J. , Keresztes, K. , Lengyel, C. , Tabák Á, G. , Spallone, V. , & Kempler, P. (2017). Why not to use the handgrip test in the assessment of cardiovascular autonomic neuropathy among patients with diabetes mellitus? Current Vascular Pharmacology, 15, 66–73. 10.2174/1570161114666160822154351 27550055

[phy270476-bib-0019] Lin, K. , Wei, L. , Huang, Z. , & Zeng, Q. (2017). Combination of Ewing test, heart rate variability, and heart rate turbulence analysis for early diagnosis of diabetic cardiac autonomic neuropathy. Medicine (Baltimore), 96, e8296. 10.1097/md.0000000000008296 29137013 PMC5690706

[phy270476-bib-0020] Madden, K. M. , Levy, W. C. , & Stratton, J. K. (2006). Exercise training and heart rate variability in older adult female subjects. Clinical and Investigative Medicine, 29, 20–28. 16553360

[phy270476-bib-0021] Moriconi, D. , Sacchetta, L. , Chiriacò, M. , Nesti, L. , Forotti, G. , Natali, A. , Solini, A. , & Tricò, D. (2023). Glomerular hyperfiltration predicts kidney function decline and mortality in type 1 and type 2 diabetes: A 21‐year longitudinal study. Diabetes Care, 46, 845–853. 10.2337/dc22-2003 36787983 PMC10090910

[phy270476-bib-0022] Oh, S. W. , Yang, J. H. , Kim, M. G. , Cho, W. Y. , & Jo, S. K. (2020). Renal hyperfiltration as a risk factor for chronic kidney disease: A health checkup cohort study. PLoS One, 15, e0238177. 10.1371/journal.pone.0238177 32881893 PMC7470278

[phy270476-bib-0023] Picard, M. , Tauveron, I. , Magdasy, S. , Benichou, T. , Bagheri, R. , Ugbolue, U. C. , Navel, V. , & Dutheil, F. (2021). Effect of exercise training on heart rate variability in type 2 diabetes mellitus patients: A systematic review and meta‐analysis. PLoS One, 16, e0251863. 10.1371/journal.pone.0251863 33999947 PMC8128270

[phy270476-bib-0024] Russell, V. , Lenth, B. B. , Buerkner, P. , Giné‐Vázquez, I. , Herve, M. , Jung, M. , Love, J. , Miguez, F. , Piaskowski, J. , Riebl, H. , & Singmann, H. (2024). Estimated Marginal Means, aka Least‐Squares Means. R package version 1.10.2.

[phy270476-bib-0025] Sarmento, A. O. , Santos, A. D. C. , Trombetta, I. C. , Dantas, M. M. , Oliveira Marques, A. C. , Do Nascimento, L. S. , Barbosa, B. T. , Dos Santos, M. R. , Andrade, M. D. A. , Jaguaribe‐Lima, A. M. , & Brasileiro‐Santos, M. D. S. (2017). Regular physical exercise improves cardiac autonomic and muscle vasodilatory responses to isometric exercise in healthy elderly. Clinical Interventions in Aging, 12, 1021–1028. 10.2147/cia.S120876 28721030 PMC5500489

[phy270476-bib-0026] Schuit, A. J. , Van Amelsvoort, L. G. , Verheij, T. C. , Rijneke, R. D. , Maan, A. C. , Swenne, C. A. , & Schouten, E. G. (1999). Exercise training and heart rate variability in older people. Medicine and Science in Sports and Exercise, 31, 816–821. 10.1097/00005768-199906000-00009 10378908

[phy270476-bib-0027] Serhiyenko, V. A. , & Serhiyenko, A. A. (2018). Cardiac autonomic neuropathy: Risk factors, diagnosis and treatment. World Journal of Diabetes, 9, 1–24. 10.4239/wjd.v9.i1.1 29359025 PMC5763036

[phy270476-bib-0028] Shields, R. W., Jr. (2009). Heart rate variability with deep breathing as a clinical test of cardiovagal function. Cleveland Clinic Journal of Medicine, 76(Suppl 2), S37–S40. 10.3949/ccjm.76.s2.08 19376980

[phy270476-bib-0029] Singmann, H. , Bolker, B. M. , & Westfall, J. (2015). Analysis of factorial experiments.

[phy270476-bib-0030] Sokolovska, J. , Ostrovska, K. , Pahirko, L. , Varblane, G. , Krilatiha, K. , Cirulnieks, A. , Folkmane, I. , Pirags, V. , Valeinis, J. , Klavina, A. , & Selavo, L. (2020). Impact of interval walking training managed through smart mobile devices on albuminuria and leptin/adiponectin ratio in patients with type 2 diabetes. Physiological Reports, 8, e14506. 10.14814/phy2.14506 32652863 PMC7354089

[phy270476-bib-0031] Spallone, V. (2019). Update on the impact, diagnosis and management of cardiovascular autonomic neuropathy in diabetes: What is defined, what is new, and what is unmet. Diabetes and Metabolism Journal, 43, 3–30. 10.4093/dmj.2018.0259 30793549 PMC6387879

[phy270476-bib-0032] Stevens, M. J. (2001). New imaging techniques for cardiovascular autonomic neuropathy: A window on the heart. Diabetes Technology & Therapeutics, 3, 9–22. 10.1089/152091501750219985 11469712

[phy270476-bib-0033] Team, R. C. (2024). R: A language and environment for statistical computing. R Foundation for Statistical Computing.

[phy270476-bib-0034] Vinik, A. I. , Maser, R. E. , Mitchell, B. D. , & Freeman, R. (2003). Diabetic autonomic neuropathy. Diabetes Care, 26, 1553–1579. 10.2337/diacare.26.5.1553 12716821

[phy270476-bib-0035] Vinik, A. I. , & Ziegler, D. (2007). Diabetic cardiovascular autonomic neuropathy. Circulation, 115, 387–397. 10.1161/circulationaha.106.634949 17242296

[phy270476-bib-0036] Voulgari, C. , Pagoni, S. , Vinik, A. , & Poirier, P. (2013). Exercise improves cardiac autonomic function in obesity and diabetes. Metabolism, 62, 609–621. 10.1016/j.metabol.2012.09.005 23084034

[phy270476-bib-0037] Ziemssen, T. , & Siepmann, T. (2019). The investigation of the cardiovascular and sudomotor autonomic nervous system—A review. Frontiers in Neurology, 10, 53. 10.3389/fneur.2019.00053 30809183 PMC6380109

