# Supplementary material for: Assessing the impact of supervised interval training on cardiovascular autonomic neuropathy in type 2 diabetes patients
Source: Physiol Rep. 2025 Aug 13;13(15):e70476. doi: 10.14814/phy2.70476 (PMC12344275; doi:10.14814/phy2.70476)
Supplement: Supplementary file 1 — Tables S1–S2. [file PHY2-13-e70476-s001.docx]

**Supplements**

**Table S 1. Characteristics of the cohort and study population distribution based on cardiovascular autonomic status.**

|  |  |  |  |  |
| --- | --- | --- | --- | --- |
| Variable | **All**  (*n* = 64) | **CAN+**  (*n* = 42) | **CAN-**  (*n* = 22) | *P*-value |
| **Demographic data** | | | | |
| Gender (male/female, n (%)) | 21/43 (33/67) | 12/30 (29/71) | 9/13 (41/59) | 0.473 |
| Age (years) | 58.62 ± 9.55 | 58.14 ± 9.45 | 59.55 ± 9.9 | 0.588 |
| Weight (kg) | 94.33 ± 18.99 | 96.69 ± 18.68 | 89.82 ± 19.2 | 0.177 |
| Body mass index (kg/m^2^) | 33.46 ± 5.48 | 34.17 ± 5.3 | 32.12 ± 5.69 | 0.169 |
| Waist/hip ratio | 0.96 ± 0.08 | 0.96 ± 0.08 | 0.97 ± 0.09 | 0.640 |
| Systolic blood pressure (mmHg) | 133.22 ± 14.43 | 134.74 ± 14.87 | 130.32 ± 13.41 | 0.234 |
| Diastolic blood pressure (mmHg) | 82 ± 7.92 | 82.62 ± 8.46 | 80.82 ± 6.79 | 0.360 |
| Arterial hypertension (n (%)) | 47 (73) | 32 (76) | 15 (68) | 0.696 |
| Heart rate (beats per minute) | 73.38 ± 7.13 | 74.38 ± 7.01 | 71.45 ± 7.12 | 0.124 |
| Duration of diabetes (years) | 6.84 ± 5.12 | 6.48 ± 4.67 | 7.55 ± 5.94 | 0.468 |
| Metabolic equivalents (MET) | 33.72 ± 25.19 | 34.92 ± 26.56 | 31.43 ± 22.77 | 0.585 |
| Non-proliferative diabetic retinopathy | 4 (6.3%) | 3 (7.1%) | 1 (4.5%) | >0.99 |
| Diabetic peripheral neuropathy | 5 (7.8%) | 4 (9.5%) | 1 (4.5%) | >0.99 |
| Diabetic nephropathy | 5 (7.8%) | 3 (7.1%) | 2 (9.1%) | >0.99 |
| Metformin | 56 (87.5%) | 38 (90.5%) | 18 (81.8%) | 0.551 |
| Sulfonylurea | 16 (25.0%) | 11 (26.2%) | 5 (22.7%) | >0.99 |
| DPP4 inhibitors | 17 (26.6%) | 10 (23.8%) | 7 (31.8%) | 0.696 |
| GLP-1 agonists | 4 (6.3%) | 3 (7.1%) | 1 (4.5%) | >0.99 |
| SGLT-2 inhibitors | 6 (9.4%) | 5 (11.9%) | 1 (4.5%) | >0.99 |
| Antihypertensive drugs | 44 (68.8%) | 31 (73.8%) | 13 (59.1%) | 0.356 |
| Statins | 19 (29.7%) | 14 (33.3%) | 5 (22.7%) | 0.553 |
| eGFR (ml/min/1,73 m^2^) | 139.14 ± 49.3 | 147.74 ± 55.82 | 122.73 ± 28.32 | **0.021** |
| HbA1c (%) | 6.87 ± 1.25 | 6.96 ± 1.26 | 6.69 ± 1.24 | 0.413 |
| Fasting glucose (mmol/L) | 7.56 ± 2.47 | 7.60 ± 2.39 | 7.48 ± 2.67 | 0.862 |
| Total cholesterol (mmol/L) | 5.29 ± 1.2 | 5.28 ± 1.11 | 5.3 ± 1.38 | 0.971 |
| HDLC (mmol/L) | 1.31 ± 0.35 | 1.3 ± 0.36 | 1.33 ± 0.34 | 0.755 |
| LDLC (mmol/L) | 3.19 ± 1.11 | 3.17 ± 1.09 | 3.22 ± 1.16 | 0.862 |
| Triglycerides (mmol/L) | 2.39 ± 3.35 | 2.62 ± 4.02 | 1.96 ± 1.33 | 0.342 |
| VO2 peak (relative, ml/kg/min) | 24.1 ± 5.1 | 23.7 ± 5.4 | 24.9 ± 4.4 | 0.348 |
| **Cardiovascular autonomic reflex tests results and Ewing score** | | | | |
| **1. Heart rate response to the Valsalva maneuver (Valsalva ratio, mm)**  • Shortest R-R interval during the Valsalva maneuver  • Longest R-R interval after the Valsalva maneuver | 1.11 ± 0.09  0.65 ± 0.08  0.72 ± 0.1 | 1.09 ± 0.07  0.64 ± 0.08  0.70 ± 0.1 | 1.15 ± 0.11  0.66 ± 0.07  0.66 ± 0.07 | **0.028**  0.301  **0.029** |
| **2. Heart rate response to standing (30s/15s ratio, mm)**  • Shortest R-R interval at the 15^th^ beat  • Longest R-R interval at the 30^th^ beat | 1.03 ± 0.06  0.73 ± 0.10  0.75 ± 0.11 | 1.01 ± 0.05  0.72 ± 0.10  0.73 ± 0.11 | 1.06 ± 0.06  0.75 ± 0.09  0.8 ± 0.09 | **0.003**  0.218  **0.008** |
| **3. Heart rate response to deep breathing (Expiration/Inspiration ratio, beats/minute)** | 12.17 ± 4.37 | 10.41 ± 3.56 | 15.45 ± 3.86 | **<0.001** |
| **4. Blood pressure response to standing (Fall in SBP, mmHg)** | 11.92 ± 11.31 | 14.81 ± 11.9 | 6.41 ± 7.67 | **0.001** |
| **5. Blood pressure response to a sustained handgrip (Rise in DBP, mmHg)** | 25.83 ± 11.92 | 25.4 3 ± 13.48 | 26.59 ± 8.4 | 0.673 |
| **Ewing score (points)** | 2.16 ± 1.01 | 2.7 ± 0.72 | 1.11 ± 0.53 | **<0.001** |

Data is presented as mean ± SD or n (%) for quantitative or categorical variables, respectively. Data in both groups was compared using the Student’s t-test for continuous variables and the *chi*-square test for categorical variables. A *P-*values < 0.05 were considered statistically significant and are highlighted in bold.

*CAN+* cardiac autonomic neuropathy positive patients, *CAN-* cardiac autonomic neuropathy negative patients, *HbA1c* glycosylated hemoglobin, *HDLC* high-density lipoprotein cholesterol, *LDLC* low-density lipoprotein cholesterol, *eGFR* estimated glomerular filtration rate, *SPB* systolic blood pressure, *DBP* diastolic blood pressure.

**Table S 2. Complete repeated measures ANOVA results for cardiovascular autonomic reflex tests and the Ewing score.**

| Cardiovascular autonomic reflex tests and Ewing score | *P*-value between control and IT groups | *P*-value between CAN- and CAN+ | *P*-value two-way between groups interaction | *P*-value within groups (intervention effect) | *P*-value two-way within-between groups interaction (control/IT vs pre/post) | *P*-value two-way within-between groups interaction (CAN-/CAN+ vs pre/post) | *P*-value three-way within-between groups interaction (control/IT *vs* CAN-/CAN+ vs pre/post |
| --- | --- | --- | --- | --- | --- | --- | --- |
| **1. Heart rate response to the Valsalva maneuver** | | | | | | | |
| Shortest R-R interval during the Valsalva maneuver | 0.342 | 0.750 | 0.600 | 0.434 | 0.703 | 0.587 | 0.395 |
| Longest R-R interval after the Valsalva maneuver | 0.772 | 0.901 | 0.812 | **0.012** | 0.222 | 0.130 | 0.572 |
| **Valsalva ratio** | 0.058 | 0.370 | 0.628 | **<0.001** | 0.158 | 0.258 | 0.629 |
| **2. Heart rate response to standing** | | | | | | | |
| Shortest R-R interval at the 15^th^ beat | 0.961 | 0.918 | 0.689 | 0.072 | 0.241 | 0.893 | 0.720 |
| Longest R-R interval at the 30^th^ beat | 0.688 | 0.955 | 0.445 | **0.004** | 0.137 | **0.017** | 0.792 |
| **30s/15s ratio** | 0.575 | 0.325 | 0.793 | **0.006** | 0.763 | **0.003** | 0.746 |
| **3. Heart rate response to deep breathing** | | | | | | | |
| **Expiration/Inspiration ratio** | 0.737 | <0.001 | 0.013 | 0.112 | 0.788 | 0.144 | 0.101 |
| **4. Blood pressure response to standing** | | | | | | | |
| SBP supine | 0.194 | 0.594 | 0.142 | 0.403 | 0.071 | 0.490 | 0.706 |
| Initial fall in SBP after standing ≤ 15 s < 3 min | 0.232 | 0.201 | 0.417 | **0.011** | 0.098 | 0.671 | 0.130 |
| Delayed fall in SBP after standing for 3 min | 0.851 | 0.308 | 0.172 | 0.311 | 0.428 | 0.388 | 0.692 |
| **Fall in SBP result** | 0.248 | **0.023** | 0.840 | 0.297 | 0.523 | 0.582 | 0.426 |
| **5. Blood pressure response to a sustained handgrip** | | | | | | | |
| Baseline DBP before a sustained handgrip | 0.364 | 0.257 | 0.731 | **0.006** | 0.251 | 0.295 | 0.751 |
| Rise in DBP during sustained handgrip after 1 min | 0.150 | 0.318 | 0.566 | 0.893 | 0.259 | 0.477 | 0.358 |
| Rise in DBP during a sustained handgrip after 2 min | 0.181 | 0.548 | **0.031** | 0.319 | 0.169 | 0.742 | 0.179 |
| Rise in DBP during a sustained handgrip after 3 min | 0.867 | 0.753 | 0.725 | 0.319 | 0.295 | 0.583 | 0.424 |
| **Rise in DBP during a sustained handgrip result** | 0.904 | 0.592 | 0.228 | 0.851 | 0.551 | 0.312 | 0.582 |
| **Ewing score** | | | | | | | |
| **Ewing score** | 0.417 | **<0.001** | 0.793 | **0.032** | 0.994 | **0.001** | 0.789 |

Results of two-way repeated measures ANOVA models adjusted for sex and duration of diabetes for cardiovascular autonomic reflex tests and their subtests. The intervention groups (control vs IT) and CAN baseline status (negative vs positive) were considered as the two main between-group effects. The effect of the intervention (pre vs post) was considered as the main within-group effect. *P-*values for three simple main effects, three two-way interactions, and one three-way interaction are reported.

*DBP* diastolic blood pressure, *SBP* systolic blood pressure, *IT* interval training group, *control* control group, *CAN+* cardiovascular autonomic positive patients, *CAN-* cardiovascular autonomic negative patients, *pre* before intervention, *post* after intervention.
